# Influence of Epoxy Resin Treatment on the Mechanical and Tribological Properties of Hemp-Fiber-Reinforced Plant-Derived Polyamide 1010 Biomass Composites

**DOI:** 10.3390/molecules26051228

**Published:** 2021-02-25

**Authors:** Maiko Morino, Tetsuto Kajiyama, Yosuke Nishitani

**Affiliations:** 1 Department of Mechanical Engineering, Graduate School of Engineering, Kogakuin University, 2665-1 Nakano, Hachioji, Tokyo 192-0015, Japan; am19057@ns.kogakuin.ac.jp; 2 Tokyo Metropolitan Industrial Technology Research Institute, 2-4-10, Aomi, Kotoku, Tokyo 135-0064, Japan; kajiyama.tetsuto@iri-tokyo.jp; 3 Department of Mechanical Engineering, Faculty of Engineering, Kogakuin University, 2665-1 Nakano, Hachioji, Tokyo 192-0015, Japan

**Keywords:** biomass polymer composites, natural fiber, surface treatment, epoxy resin treatment, tribology, mechanical properties, hemp fiber, plant-derived polyamide

## Abstract

In this study, we investigated the influence of epoxy resin treatment on the mechanical and tribological properties of hemp fiber (HF)-reinforced plant-derived polyamide 1010 (PA1010) biomass composites. HFs were surface-treated using four types of surface treatment methods: (a) alkaline treatment using sodium chlorite (NaClO_2_) solution, (b) surface treatment using epoxy resin (EP) solution after NaClO_2_ alkaline treatment, (c) surface treatment using an ureidosilane coupling agent after NaClO_2_ alkaline treatment (NaClO_2_ + A-1160), and (d) surface treatment using epoxy resin solution after the (c) surface treatment (NaClO_2_ + A-1160 + EP). The HF/PA1010 biomass composites were extruded using a twin-screw extruder and injection-molded. Their mechanical properties, such as tensile, bending, and dynamic mechanical properties, and tribological properties were evaluated by the ring-on-plate-type sliding wear test. The strength, modulus, specific wear rate, and limiting *pv* value of HF/PA1010 biomass composites improved with surface treatment using epoxy resin (NaClO_2_ + A-1160 + EP). In particular, the bending modulus of NaClO_2_ + A-1160 + EP improved by 48% more than that of NaClO_2_, and the specific wear rate of NaClO_2_ + A-1160 + EP was one-third that of NaClO_2_. This may be attributed to the change in the internal microstructure of the composites, such as the interfacial interaction between HF and PA1010 and fiber dispersion. As a result, the mode of friction and wear mechanism of these biomass composites also changed.

## 1. Introduction

Natural-fiber-reinforced biomass polymer composites are attracting extensive interest as a solution for growing environmental threats [1,2,3,4,5,6,7,8,9,10,11,12]. Owing to their unique characteristics, namely eco-friendliness, light weight, low cost, and high relative strength, these biomass composites have gradually been applied to engineering materials in various industrial applications, such as structural materials and tribomaterials for mechanical sliding parts, such as bearing, cum, gear, and seal, in recent years [12,13,14,15,16]. In particular, to further enhance the eco-friendliness of these biomass polymer composites, fully bio-based polymers such as polylactic acid (PLA) [17,18,19,20,21,22] and plant-derived polyamide (PA1010, PA11) [12,23,24,25] should ideally be used as the matrix polymer. However, these biomass composites have such drawbacks as poor interfacial adhesion between natural fiber and matrix biopolymer [3,12,14,26] and may, therefore, show poor mechanical and tribological properties. Consequently, there is a need to control the interfacial adhesion between natural fiber and matrix polymer in order to further enhance the mechanical and tribological properties of these biomass composites. Specifically, chemical and physical methods for the surface modification of natural fibers can be used to optimize the interface between fiber and polymer. For example, chemical methods, such as alkaline treatment (mercerization) [27], acetylation [28], silane coupling treatment [12,14,24], isocyanate treatment [29], graft copolymerization [30], and maleic acid treatment [31], and physical treatments, such as corona treatment [32], plasma treatment [33], and γ-ray treatment [34], have been investigated for use for these composites. Considerable attention has been given to the effect of the modification of the natural fiber by chemical and physical methods on the mechanical properties of these biomass composites. In addition, there has been interest in the effects of the surface treatment of natural fiber on the tribological properties of these biomass composites [12,14,35,36,37,38,39,40,41,42,43,44].

In our previous studies, we developed new polymeric tribomaterials made of 100% inedible plant-derived materials. In particular, we investigated the effects of surface treatment on the thermal, rheological, mechanical, and tribological properties of hemp fiber (HF)-reinforced plant-derived polyamide 1010 (PA1010) biomass composites (HF/PA1010) [12,24,45,46,47,48,49,50], which are new polymeric tribomaterials made of 100% inedible plant-derived materials we developed. We found that the thermal, rheological, mechanical, and tribological properties of HF/PA1010 biomass composites improved with HF surface treatment combining alkaline treatment and the use of a silane coupling agent. In particular, the mechanical and tribological properties of these HF/PA1010 biomass composites improved with surface treatment combining alkaline treatment using sodium hydroxide (NaOH) or sodium chlorite (NaClO_2_) solution and the use of an ureidosilane coupling agent (A-1160) [12,48]. This improvement may be attributed to the change in the internal microstructure of the composites, such as the interfacial interaction between HF and PA1010 and fiber dispersion.

However, to further enhance the mechanical and tribological properties of these biomass composites, it is essential to understand the influence of the surface treatment of natural fibers other than the influence of combining alkaline treatment and the use of an ureidosilane coupling agent on the mechanical and tribological properties of these biomass composites. In this study, we propose a new surface treatment method using epoxy resin for improving mechanical properties, such as strength and modulus, and tribological properties, such as specific wear rate and limiting *pv* value. This epoxy resin treatment is expected to generate hydrogen bonding between the epoxy groups in the epoxy resin and the ureido groups in the ureidosilane coupling agent on the surface of alkaline-treated HF, as well as generate chemical reactions between the epoxy groups in the epoxy resin and highly reactive functional groups such as amine and carboxyl end groups in the molecular chain of polyamides.

To develop new polymeric engineering materials made only from inedible plant-derived materials, this study aimed to experimentally investigate the influence of epoxy resin treatment on the mechanical and tribological properties of hemp-fiber-reinforced plant-derived PA1010 biomass composites. Four types of surface treatment of HF were carried out: (a) alkaline treatment using sodium chlorite (NaClO_2_) solution, (b) surface treatment using epoxy resin (EP) solution after NaClO_2_ alkaline treatment (NaClO_2_ + EP), (c) surface treatment using an ureidosilane coupling agent after NaClO_2_ treatment (NaClO_2_ + A-1160), and (d) surface treatment using epoxy resin solution after the (c) surface treatment (NaClO_2_ + A-1160 + EP).

## 2. Results and Discussion

### 2.1. Fourier-Transform Infrared (FT-IR) Analysis of Fiber Surface

**Figure 1:**
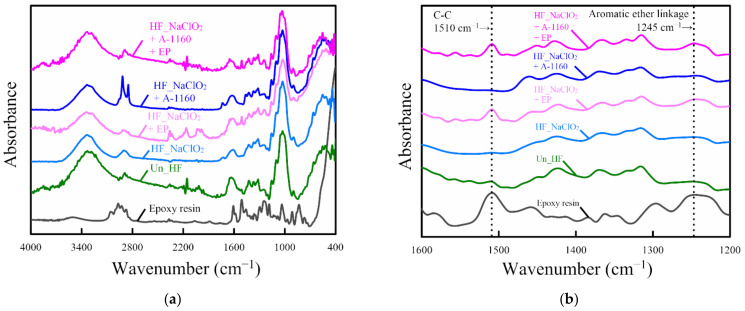
Fourier-transform infrared (FT-IR) spectrum of various surface-treated hemp fibers: (**a**) peak located at 400–4000 cm^−1^ and (**b**) peak located at 1200–1600 cm^−1^.

This section discusses the chemical composition of the surface-treated hemp fiber (HF) using Fourier-transform infrared (FT-IR) spectroscopy. Figure 1 shows the FT-IR spectra of epoxy resin and various surface-treated hemp fibers where the peaks were located at 400–4000 cm^−1^ (Figure 1a) and 1200–1600 cm^−1^ (Figure 1b). In the FT-IR spectrum of the untreated hemp fiber (Un_HF), some typical peaks were observed at 1162 cm^−1^ due to the C-O-C asymmetrical stretching of cellulose and hemicellulose; at 1400–1500 cm^−1^ due to the CH_2_ bending of pectin, lignin, and hemicellulose; at 1616 cm^−1^ due to the benzene ring stretching of lignin; at 1730 cm^−1^ due to C=O stretching in hemicellulose; at 2850–2950 cm^−1^ due to the CH_2_ stretching of wax and the C-H stretching of polysaccharides; and at 3200–3600 cm^−1^ due to the OH stretching of polysaccharides [14,24,51,52,53]. As reported in a previous paper [24], peaks at 1400–1500, 1616, 1730, 2850–2950, and 3200–3600 cm^−1^ disappear with NaClO_2_ alkaline treatment. Alkaline treatment using NaClO_2_ has more attackability on HF, which is able to remove lignin, wax, and hemicellulose from HF bundles and replaces more OH groups on the surface of HF than that using sodium hydroxide (NaOH). On the contrary, the FT-IR spectra of surface treatment using an ureidosilane coupling agent (A-1160) show some peaks that are at 1730 cm^−1^ due to C=O stretching, at 2850 and 2910 cm^−1^ due to CH_2_ stretching, and at 3200–3600 cm^−1^ due to OH stretching. Accordingly, the peaks at 1710, 2850, and 2910 cm^−1^ show the presence of silane on the surface when surface treatment is performed using A-1160. These peaks are not present when only NaClO_2_ alkaline treatment is performed. Incidentally, Sawpan [53], Valadez-Gonzalez [54], and Pothan [55] reported the presence of the ureidosilane coupling agent on the fiber, which showed peaks at 708 cm^−1^ due to -Si-O-Si- symmetric stretching, at 780 cm^−1^ due to -Si-C- symmetric stretching, and at 1203 cm^−1^ due to –Si-O-C- stretching. However, as reported by Sgriccia [52], the concentration of the silane coupling agent on the fiber surface is too small to be detected by FT-IR in this study.

The FT-IR spectrum of epoxy resin based on bisphenol A shows some clear peaks. In particular, there are two large, clear peaks in this spectrum. One is a peak at 1245 cm^−1^, which is caused by the antisymmetric stretching vibration of the aromatic ether linkage, and the other is a peak at 1510 cm^−1^, which is caused by the C-C stretching of the benzene ring [56]. On the other hand, these peaks can also be observed in the use of HF_NaClO_2_ + EP and HF_NaClO_2_ + A-1160 + EP, suggesting the presence of epoxy resin due to the surface treatment of HF by epoxy resin (EP). These peaks are not seen in surface treatments that do not use epoxy resin (HF_NaClO_2_ and HF_NaClO_2_ + A-1160), indicating that the surface treatment using epoxy resin is effective for HF after NaClO_2_ alkaline treatment or after surface treatment using NaClO_2_ alkaline and an ureidosilane coupling agent (A-1160) together.

### 2.2. Morphology of HF/PA1010 Biomass Composites

**Figure 2:**
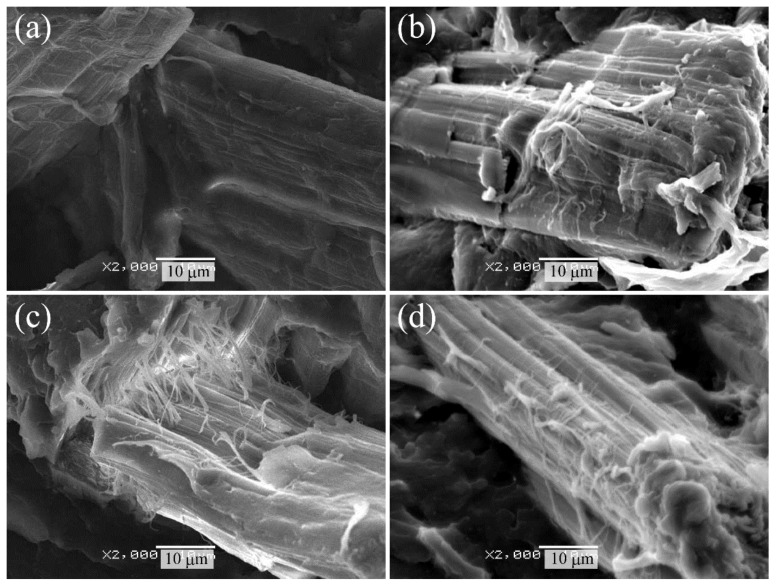
Scanning electron microscope (SEM) photographs of the cryogenically fractured surface of various surface-treated hemp-fiber-reinforced plant-derived polyamide 1010 (HF/PA1010) biomass composites: (**a**) HF_NaClO_2_, (**b**) HF_NaClO_2_ + EP, (**c**) HF_NaClO_2_ + A-1160, and (**d**) HF_NaClO_2_ + A-1160 + EP (×2000).

This section discusses the morphologies of various surface-treated HF/PA1010 biomass composites to clarify the internal microstructures of these biomass composites, such as the interfacial interaction between HF and PA1010 and fiber dispersion in these biomass composites. We carried out the scanning electron microscope (SEM) observation of the fractured surface cryogenically in liquid nitrogen. Figure 2 shows the SEM photographs of cryogenically fractured surfaces of various surface-treated HF/PA1010 biomass composites: HF_NaClO_2_ (Figure 2a), HF_NaClO_2_ + EP (Figure 2b), HF_NaClO_2_ + A-1160 (Figure 2c), and HF_NaClO_2_ + A-1160 + EP (Figure 2d). In terms of morphology, HF_NaClO_2_ (Figure 2a) had a relatively clean and smooth fiber surface, suggesting poor chemical and physical interaction between HF and PA1010. On the other hand, the morphologies of HF_NaClO_2_ +EP (Figure 2b), HF_NaClO_2_ + A-1160 (Figure 2c), and HF_NaClO_2_ + A-1160 + EP (Figure 2d) demonstrated good interaction between HF and PA1010, and there were no voids at the interfaces between the fibers and the matrix polymer. In particular, there was more residual polymer on the fiber surface of HF_NaClO_2_ + A-1160 + EP (Figure 2d) than that of HF_NaClO_2_ + EP (Figure 2b) and that of HF_NaClO_2_ + A-1160 (Figure 2c). Thus, these results are supported by the fact that surface treatment using epoxy resin after combining NaClO_2_ alkaline treatment and the use of an ureidosilane coupling agent (HF_NaClO_2_ + A-1160 + EP) generates good interfacial interaction between HF and PA1010. This may be due to chemical reactions among the epoxy groups in the epoxy resin, the ureido groups in the ureidosilane coupling agent on the alkaline-treated HF, and the highly reactive functional groups such as amine and carboxyl end groups in the molecular chain of polyamide.

**Figure 3:**
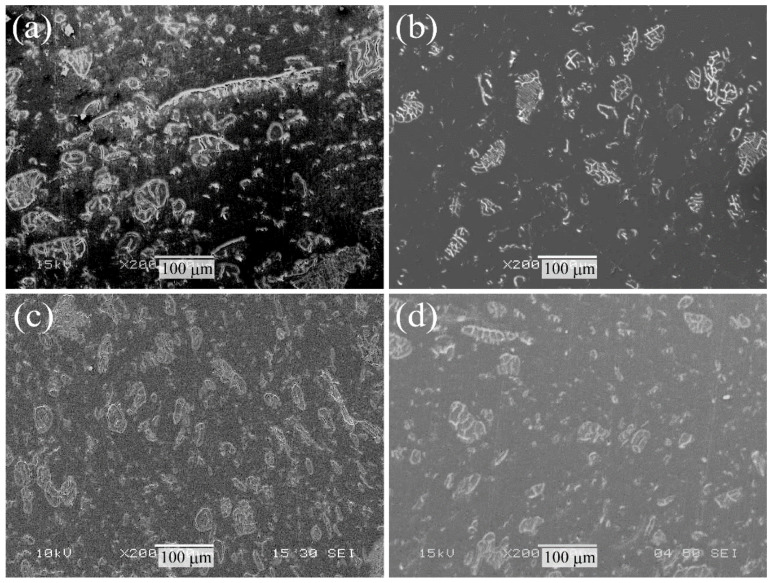
SEM photographs of the polished cross-sectional surface of various surface-treated HF/PA1010 biomass composites: (**a**) HF_NaClO_2_, (**b**) HF_NaClO_2_ + EP, (**c**) HF_NaClO_2_ + A-1160, and (**d**) HF_NaClO_2_ + A-1160 + EP (×200).

Next, Figure 3 shows the SEM photographs of the polished cross-sectional surface of various surface-treated HF/PA1010 biomass composites: HF_NaClO_2_ (Figure 3a), HF_NaClO_2_ + EP (Figure 3b), HF_NaClO_2_ + A-1160 (Figure 3c), and HF_NaClO_2_ + A-1160 + EP (Figure 3d). These SEM photographs show the differences in the fiber dispersion in the composites. The dispersion of HF in the composites improved with some types of surface treatments in the following order: HF_NaClO_2_ (Figure 3a) < HF_NaClO_2_ + EP (Figure 3b) < HF_NaClO_2_ + A-1160 (Figure 3c) < HF_NaClO_2_ + A-1160 + EP (Figure 3d). In particular, individual separation and good dispersion of HF were seen in the morphology of the polished cross-sectional surface of HF_NaClO_2_ + A-1160 + EP (Figure 3d). There was also no agglomeration of fibers, which showed that surface treatment using epoxy resin after surface treatment by combining NaClO_2_ alkaline treatment and the use of A-1160 improves the dispersion of fibers. This dispersion of HF is related to the change in the interfacial interaction between HF and PA1010 when melt-mixing using a twin-screw extruder according to the surface treatment of HF is performed. Especially, surface treatment using epoxy resin after surface treatment combining NaClO_2_ alkaline treatment and the use of A-1160 (NaClO_2_ + A-1160 + EP) is expected to cause stronger interfacial interaction between HF and PA1010 than other surface treatments.

### 2.3. Thermogravimetric Analysis

**Figure 4:**
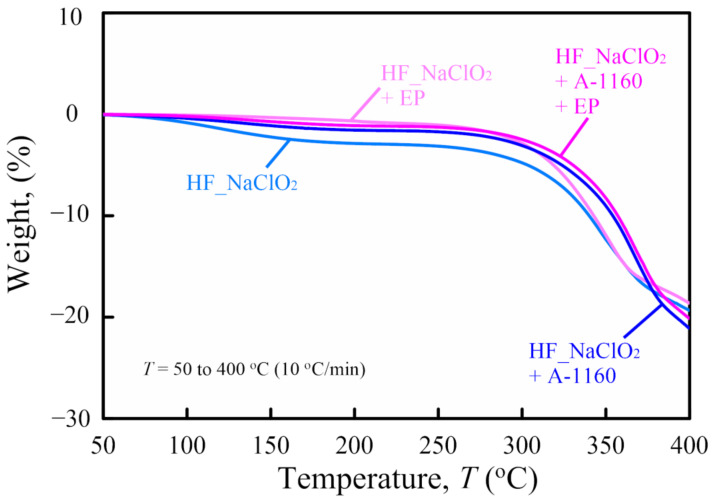
Weight as a function of temperature *T* (thermogravimetric (TG) curve) of various Scheme 1010. biomass composites.

To evaluate the influence of surface treatment on the thermal stability of various surface-treated HF/PA1010 biomass composites, thermogravimetric analysis (TGA) of these biomass composites is discussed in this section. Figure 4 shows weight as a function of temperature *T* (TG curve) of various surface-treated HF/PA1010 biomass composites. As reported in our previous paper [24], the TG curves of various surface-treated HF/PA1010 biomass composites provide evidence of two types of weight loss. The first weight loss occurs between 80 °C and 200 °C due to the dehydration of HF as well as the thermal degradation of lignin and hemicellulose [14,24]. In particular, the first weight loss of HF_NaClO_2_ was the greatest in the various surface-treated HF/PA1010 biomass composites used in this study. Although alkaline treatment using NaClO_2_ leaves some HF sub-component residues such as lignin and hemicellulose, surface treatments using an ureidosilane coupling agent (A-1160) or epoxy resin covered the HF with a coating and inhibited the thermal degradation of lignin and hemicellulose. On the other hand, the second weight loss occurred at more than 300 °C due to the decomposition of cellulose in HF. The details of the temperature in the case of 10 and 15 wt % loss of various surface-treated HF/PA1010 biomass composites are listed in Table 1. The temperature at 10 and 15 wt % of various surface-treated HF/PA1010 biomass composites increased in the following order: HF_NaClO_2_ < HF_NaClO_2_ + EP < HF_NaClO_2_ + A-1160 < HF_NaClO_2_ + A-1160 + EP. These behaviors may be attributed to the fact that HFs are protected by A-1160 surface treatment.

Lu et al. reported similar tendencies, for example, the thermal stability of silane-treated hemp-fiber-reinforced polyethylene composites is higher than that of untreated and alkaline-treated ones [57]. In particular, they concluded that not only does silane treatment modify the surface of hemp but when silane-treated fibers come in contact with the polymer matrix, the organo-functional group of the silane molecule couples with the matrix and increases the strength of bonding. Moreover, the enhanced covalent bonding along with the physical compatibility with the polymer matrix increases the thermal stability of the resultant composites.

Therefore, in this study, surface treatment using an ureidosilane coupling agent (A-1160) increased the interfacial interaction between HF and PA1010 and was thermally more stable than that without interfacial interaction generated by A-1160. These results indicate why pre-treatment combining NaClO_2_ alkaline treatment and the use of A-1160 is essential before surface treatment using epoxy resin.

### 2.4. Mechanical Properties

**Figure 5:**
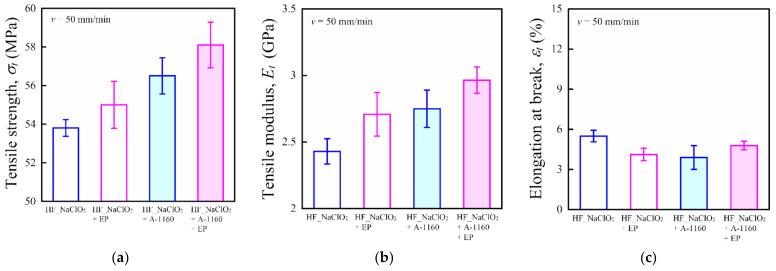
Tensile properties of various surface-treated HF/PA1010 biomass composites: (**a**) tensile strength *σ_t_*, (**b**) tensile modulus *E_t_*, and (**c**) elongation at break *ε_t_*.

This section discusses the influence of epoxy resin treatment on mechanical properties, such as tensile, bending, and dynamic mechanical properties, of HF/PA1010 biomass composites. Figure 5 shows the tensile properties, namely tensile strength *σ_t_* (Figure 5a), tensile modulus *E_t_* (Figure 5b), and elongation at break *ε_t_* (Figure 5c), of various surface-treated HF/PA1010 biomass composites. The *σ_t_* and *E_t_* of HF/PA1010 biomass composites improved with the surface treatment of the fiber using an ureidosilane coupling agent (A-1160) or epoxy resin (EP), although the improvement effects differed according to the type of fiber surface treatment performed. The *σ_t_* and *E_t_* of various surface-treated HF/PA1010 biomass composites increased in the following order: HF_NaClO_2_ < HF_NaClO_2_ + EP < HF_NaClO_2_ + A-1160 < HF_NaClO_2_ + A-1160 + EP. On the other hand, the *ε_t_* of all HF/PA1010 biomass composites was more or less the same level, although the values of *ε_t_* differed slightly according to the type of fiber surface treatment performed. 

**Figure 6:**
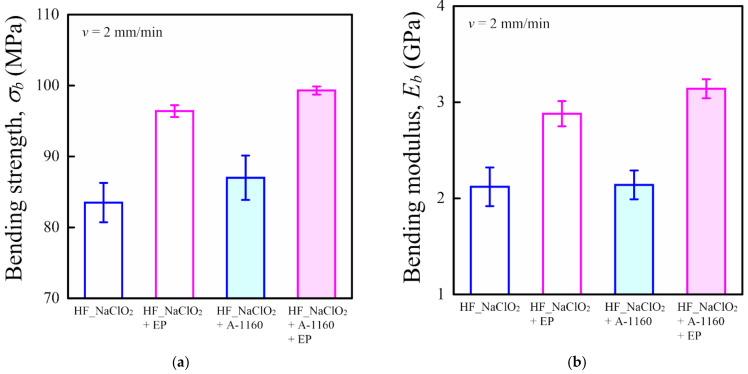
Bending properties of various surface-treated HF/PA1010 biomass composites: (**a**) bending strength *σ_b_* and (**b**) bending modulus *E_b_*.

Figure 6 shows the bending properties, such as bending strength *σ_b_* (Figure 6a) and bending modulus *E_b_* (Figure 6b), of various surface-treated HF/PA1010 biomass composites. The *σ_b_* and *E_b_* of various surface-treated HF/PA1010 biomass composites had different tendencies from tensile properties and increased in the following order: HF_NaClO_2_ < HF_NaClO_2_ + A-1160 < HF_NaClO_2_ + EP < HF_NaClO_2_ + A-1160 + EP. In particular, the *σ_b_* and *E_b_* of HF_NaClO_2_ + A-1160 + EP significantly improved compared to those of the other surface-treated HF/PA1010 biomass composites. The results for these static mechanical properties, such as tensile and bending properties, indicated that surface treatment using epoxy resin after pretreatment combining NaClO_2_ alkaline treatment and the use of an ureidosilane coupling agent (NaClO_2_ + A-1160) is the most effective surface treatment for enhancing mechanical properties such as the strength and rigidity, of HF/PA1010 biomass composites. This may be due to the change in the interfacial interactions between HF and PA1010 according to the type of surface treatment performed. 

Specifically, these interfacial interactions are chemical reactions and physical interactions between HF and PA1010 surface-treated by HF_NaClO_2_ + A-1160 + EP based on the following mechanisms:

1. NaClO_2_ alkaline treatment removes sub-components such as lignin, wax, and hemicellulose from the HF surface, increases the surface roughness, and, moreover, generates hydroxyl groups.
2. The silane coupling agent generates alkoxysilane groups, which after hydrolysis are capable of reacting with OH-rich fiber surfaces [58]. Therefore, after HF is treated by NaClO_2_, the condensation reaction between the silanol groups of the ureidosilane coupling agent (A-1160) and the hydroxyl groups on the surface of HF can occur more easily.
3. Ureido groups in A-1160 can cause hydrogen bonding with hydroxyl groups and epoxy groups in epoxy resin. This reinforces the interfacial interaction between A-1160 and epoxy resin, enhancing the compatibility between two phases.
4. Chemical reactions between epoxy groups in epoxy resin and highly reactive functional groups such as amine and carboxyl end groups in the molecular chain of polyamides can occur more easily.

Therefore, we found that epoxy resin treatment significantly improves mechanical properties such as strength and modulus by varying the interfacial interaction between HF and PA1010 based on the mechanisms mentioned above. On the other hand, the difference in the effects of surface treatment on tensile and bending test results is due to the different deformation modes in each test. Specifically, while the tensile stress against the force applied in the same direction as the strain is measured in the tensile test, the bending stress, which is a complex stress consisting of tensile and compressive stress against a force generated by the three-point bending method, is measured in the bending test. In addition, different test speeds and test geometries (thickness) were used in the two tests. The tensile and bending test results showed different tendencies, but it is difficult to determine the detailed reasons at this point.

**Figure 7:**
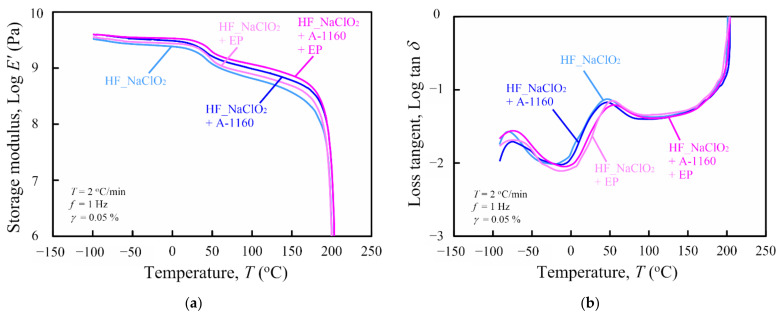
Dynamic mechanical analysis (DMA) curves as a function of temperature for various HF/PA1010 biomass composites: (**a**) storage modulus *E’* and (**b**) loss tangent tan*δ*.

The dynamic viscoelastic properties of various surface-treated HF/PA1010 biomass composites in the solid state were determined by dynamic mechanical analysis (DMA) experiments. The storage modulus *E’* and the loss tangent tan*δ* (=loss modulus *E”*/storage modulus *E’*) were plotted as functions of temperature *T* of various surface-treated HF/PA1010 biomass composites, as shown in Figure 7a (*E’* vs. *T*) and Figure 7b (tan*δ* vs. *T*), respectively. The *E’* of HF_NaClO_2_ + A-1160 + EP was the highest among the storage moduli of all surface-treated HF/PA1010 biomass composites in all temperature regions. Thus, a higher *E’* indicates a higher mechanical rigidity of HF_NaClO_2_ + A-1160 + EP than other surface-treated HF/PA1010 biomass composites. There was a significant decrease in the temperature in the regions between 40 °C and 70 °C, which is related to the glass transition temperature *T_g_* of PA1010. These softening temperatures shifted slightly toward a high temperature according to the type of fiber surface treatment carried out and increased in the following order: HF_NaClO_2_ < HF_NaClO_2_ + EP < HF_NaClO_2_ + A-1160 < HF_NaClO_2_ + A-1160 + EP. These shifts may be attributed to the change in the molecular chain mobility in the amorphous segment of the matrix polymer according to the surface treatment using epoxy resin. Various studies on the DMA of surface-treated natural-fiber-reinforced biomass polymer composites have reported these shifts according to whether surface treatment was used or not used [18,24,59,60,61,62]. On the other hand, the tan*δ* curves in Figure 7b have two relaxation peaks. The first peak, between 40 °C and 70 °C, indicates *α* relaxation, which is related to the *T_g_* of PA1010 and the composites mentioned earlier. On the contrary, the second peak, between −90 °C and −60 °C, indicates the relaxation of the hydrogen bonds between the PA1010 chains [24,63]. This second peak in the lower-temperature region shifted slightly toward higher temperatures with the surface treatment of fibers, due to restricted movement of the polymer chains.

From the results of the comparison of static and dynamic mechanical properties of various surface-treated HF/PA1010 biomass composites, it can be concluded that surface treatment using epoxy resin after pre-treatment by NaClO_2_ alkaline treatment and the use of an ureidosilane coupling agent (HF_NaClO_2_ + A-1160 + EP) is the most effective surface treatment method for enhancing the dynamic mechanical properties of these biomass composites. These findings are consistent with the observed morphologies of composites, such as SEM observations of cryogenically fractured surfaces (Figure 2) and cross-sectional polished surfaces (Figure 3), as mentioned earlier. The high strength and modulus of HF/PA1010 biomass composites with surface treatment using epoxy resin are considered to be due to the change in the interfacial interaction between HF and PA1010 and the morphological change in the fiber dispersion in the composites.

### 2.5. Tribological Properties

**Figure 8:**
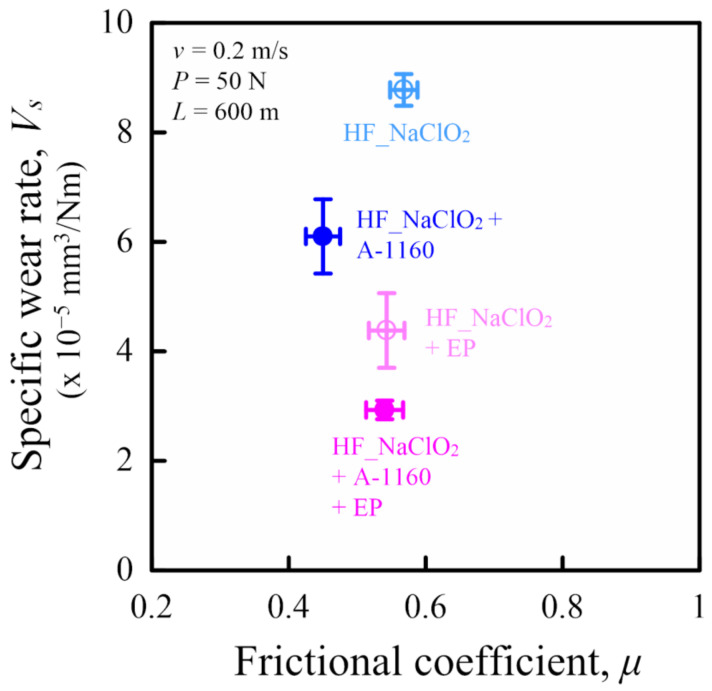
Relationship between specific wear rate and frictional coefficient of various surface-treated HF/PA1010 biomass composites using a ring-on-plate-type sliding wear test (*P* = 50 N, *v* = 0.2 m/s, and *L* = 600 m).

This section discusses the influence of surface treatment using epoxy resin on tribological properties using a ring-on-plate-type sliding wear tester under constant load and constant velocity and under dry conditions of HF/PA1010 biomass composites against the metal counterpart (carbon steel S45C). Figure 8 shows the relationship between the frictional coefficient *µ* and the specific wear rate *V_s_* of various surface-treated HF/PA1010 biomass composites. The test conditions were a constant load *P* of 50 N, a constant velocity *v* of 0.2 m/s, and a sliding distance *L* of 600 m. *µ* was calculated as the average value from 400 m to 600 m, and *V_s_* was calculated as the difference in the mass loss of the plate specimens made of polymer composites before and after testing. In this figure, friction and wear decreased toward the bottom left, indicating that high performance is required for application to tribomaterials. The *µ* of various surface-treated HF/PA1010 biomass composites decreased in the following order: HF_NaClO_2_ > HF_NaClO_2_ + EP > HF_NaClO_2_ + A-1160 + EP > HF_NaClO_2_ + A-1160. On the other hand, the *V_s_* of these biomass composites decreased in the following order: HF_NaClO_2_ > HF_NaClO_2_ + A-1160 > HF_NaClO_2_ + EP > HF_NaClO_2_ + A-1160 + EP. Thus, the *V_s_* of the composites has different tendencies from that of *µ*. These results may be attributed to the change in the mode of friction and wear mechanism according to the type of surface treatment of HF. It is rational to conclude that the tribological properties of these biomass composites are strongly influenced by the surface treatment of the fiber. In particular, the wear properties of these biomass composites notably improve with surface treatment using epoxy resin. The tribological behavior of polymer composites is strongly influenced by their ability to form transfer films on the metallic counterface and by the worn surfaces of materials and wear debris. Thus, it is essential to study these factors to understand the friction and wear mechanisms of the polymer composites [12,64,65].

**Figure 9:**
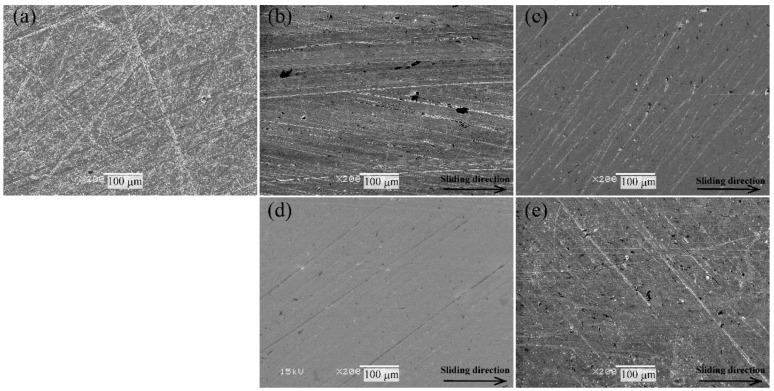
SEM photographs of metallic counterface (carbon steel S45C) before and after sliding wear tests of various surface-treated HF/PA1010 biomass composites: (**a**) surface before sliding, (**b**) surface after sliding for HF_NaClO_2_, (**c**) surface after sliding for HF_NaClO_2_ + EP, (**d**) surface after sliding for HF_NaClO_2_ + A-1160, and (**e**) surface after sliding for HF_NaClO_2_ + A-1160 + EP) (×200).

Figure 9 shows the SEM photographs of the metallic counterface (carbon steel S45C) before and after sliding wear tests of various surface-treated HF/PA1010 biomass composites. Specifically, Figure 9a shows the surface before sliding, Figure 9b shows the surface after sliding for HF_NaClO_2_, Figure 9c for HF_NaClO_2_ + EP, Figure 9d for HF_NaClO_2_ + A-1160, and Figure 9e for HF_NaClO_2_ + A-1160 + EP. After the sliding wear tests, the metallic counterfaces showed differences in the polymeric transfer films formed on the counterfaces according to the type of HF surface treatment carried out. These polymeric transfer films are thin and have smooth surfaces. The apparent thickness of the transfer film can be determined from the appearance of the scratch marks left by the polishing paper on the metallic counterface to keep the surface roughness (*R_a_* = 0.4 µm) constant before the sliding wear tests. Specifically, the thinner these transfer films are, the clearer are the scratch marks on the metallic counterface; the thicker these films are, the less obvious are the scratches. Their thickness decreased in the following order: HF_NaClO_2_ (Figure 9b) > HF_NaClO_2_ + EP (Figure 9c) > HF_NaClO_2_ + A-1160 + EP (Figure 9e) > HF_NaClO_2_ + A-1160 (Figure 9d). Small, thin scratch marks could also be seen on the whole surface of the counterfaces for all the biomass composites. These scratches were found to keep the surface roughness constant before the sliding wear tests (Figure 9a). Moreover, there was also considerable debris on the counterfaces of HF_NaClO_2_ (Figure 9b), HF_NaClO_2_ + EP (Figure 9c), and HF_NaClO_2_ + A-1160 + EP (Figure 9e) because these biomass composites have relatively high frictional coefficients. On the contrary, there was no wear debris on the counterface of the HF_NaClO_2_ + A-1160 biomass composite (Figure 9d), which has a low *µ*.

**Figure 10:**
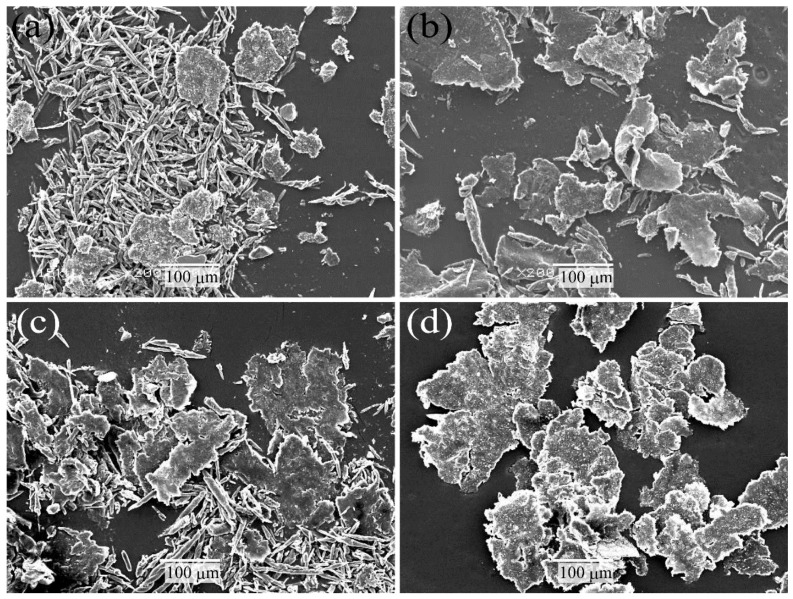
SEM photographs of wear debris after sliding wear tests of various surface-treated HF/PA1010 biomass composites: (**a**) HF_NaClO_2_, (**b**) HF_NaClO_2_ + EP, (**c**) HF_NaClO_2_ + A-1160, and (**d**) HF_NaClO_2_ + A-1160 + EP (×200).

Figure 10 shows SEM photographs of wear debris after the sliding wear tests of various surface-treated HF/PA1010 biomass composites: HF_NaClO_2_ (Figure 10a), HF_NaClO_2_ + EP (Figure 10b), HF_NaClO_2_ + A-1160 (Figure 10c), and HF_NaClO_2_ + A-1160 + EP (Figure 10d). The wear debris of various surface-treated HF/PA1010 biomass composites was collected from the sliding surface after the sliding wear test. The shape and size of the wear debris differed according to the type of surface treatment of HF. The debris of HF_NaClO_2_ (Figure 10a) was a mixture of many filaments and some big, flaky particles. The debris of HF_NaClO_2_ + A-1160 (Figure 10c) was a mixture of small filaments and many big, flaky particles. On the contrary, the debris of HF_NaClO_2_ + EP (Figure 10b) had more flaky particles but less filamentary ones. Moreover, the debris of HF_NaClO_2_ + A-1160 + EP (Figure 10d) consisted of only big and flaky particles, without filamentary ones. Therefore, the shape of the wear debris of HF/PA1010 biomass composites changed from filamentary ones to flaky ones when surface treatment was performed using an ureidosilane coupling agent and epoxy resin. Furthermore, the size of the wear debris of these surface-treated biomass composites increased to larger flaky particles than those of HF_NaClO_2_. These differences may support the assumption that the change in the mode of friction and wear mechanism according to the type of surface treatment of HF is caused by good interfacial interaction between HF and PA1010 and good dispersion of fiber in the composites.

**Figure 11:**
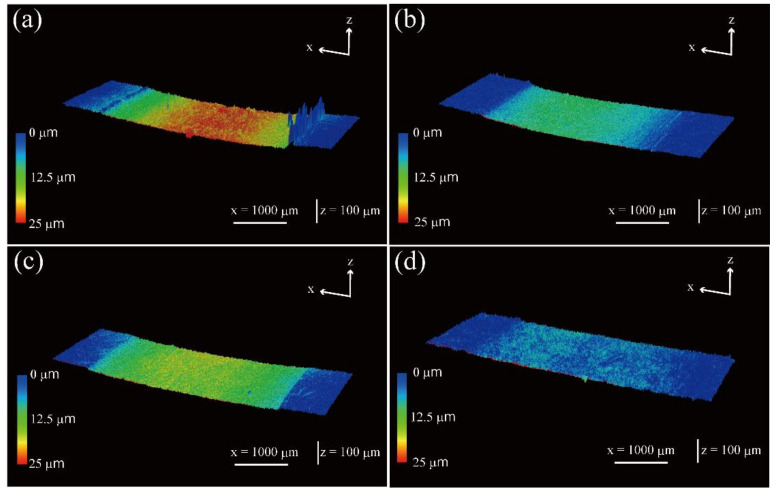
Three-dimensional profiles of the worn surface of polymer composite specimens after the sliding wear test against carbon steel S45C of various surface-treated HF/PA1010 biomass composites: (**a**) HF_NaClO_2_, (**b**) HF_NaClO_2_ + EP, (**c**) HF_NaClO_2_ + A-1160, and (**d**) HF_NaClO_2_ + A-1160 + EP.

**Figure 12:**
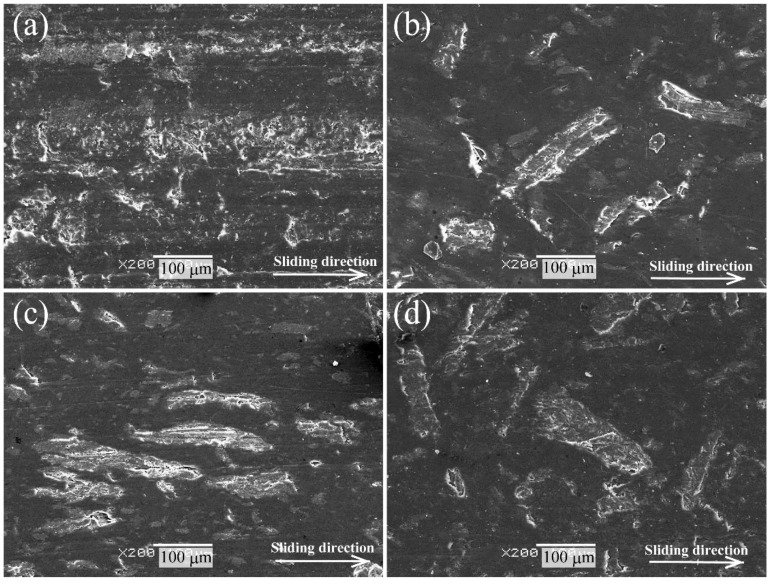
SEM photographs of the worn surface of polymer composite specimens after the sliding wear test against carbon steel S45C of various surface-treated HF/PA1010 biomass composites: (**a**) HF_NaClO_2_, (**b**) HF_NaClO_2_ + EP, (**c**) HF_NaClO_2_ + A-1160, and (**d**) HF_NaClO_2_ + A-1160 + EP (×200).

Figure 11 shows the three-dimensional profiles of the worn surfaces of polymer composite specimens after the sliding wear tests of the carbon steel S45C of various surface-treated HF/PA1010 biomass composites: HF_NaClO_2_ (Figure 11a), HF_NaClO_2_ + EP (Figure 11b), HF_NaClO_2_ + A-1160 (Figure 11c), and HF_NaClO_2_ + A-1160 + EP (Figure 11d). The depth of the wear tracks and the roughness of the worn surface of these biomass composites decreased in the following order: HF_NaClO_2_ (Figure 11a) > HF_NaClO_2_ + A-1160 (Figure 11c) > HF_NaClO_2_ + EP (Figure 11c) > HF_NaClO_2_ + A-1160 + EP (Figure 11d). Figure 12 shows the SEM photographs of the worn surface of biomass polymer composites: HF_NaClO_2_ (Figure 12a), HF_NaClO_2_ + EP (Figure 12b), HF_NaClO_2_ + A-1160 (Figure 12c), and HF_NaClO_2_ + A-1160 + EP (Figure 12d). The morphologies of the worn surfaces of these biomass composites changed according to the type of surface treatment of HF. The worn surfaces of HF_NaClO_2_ (Figure 11a and Figure 12a) had very rough and bumpy faces. In particular, there were two high-wear walls, where the wear debris accumulated, at the boundary between the worn surface and the non-worn surface (Figure 11a). Moreover, there were countless small wear-and-tear marks, and HF could not be clearly seen on the worn surface of HF_NaClO_2_ (Figure 12a). On the other hand, the worn surfaces of HF_NaClO_2_ + A-1160 (Figure 11c and Figure 12c) were smoother than that of HF_NaClO_2_. HF could be clearly seen on the surface of HF_NaClO_2_ + A-1160, although the HFs in HF_NaClO_2_ + A-1160 were damaged and the surfaces showed many frictional marks (Figure 12c). The worn surface of HF_NaClO_2_ + A-1160 showed slight fiber debonding with PA1010, which is a gap in the interface between HF and PA1010. On the contrary, the worn surfaces of HF_NaClO_2_ + EP (Figure 11b and Figure 12b) and HF_NaClO_2_ + A-1160 + EP (Figure 11d and Figure 12d) showed very smooth surfaces. In particular, HF could also be clearly seen on the surface of HF_NaClO_2_ + A-1160 + EP, and it was not severely damaged. Especially, the fibers of HF_NaClO_2_ + A-1160 + EP did bond with PA1010 and fiber breakage occurred. These differences may be due to the change in the mode of friction and wear mechanism according to the type of surface treatment of HF carried out or due to improvement in mechanical properties as a result of the strong interfacial interaction between HF and PA1010 and good fiber dispersion in the composites in surface treatment using epoxy resin after pre-treatment by NaClO_2_ alkaline treatment and the use of an ureidosilane coupling agent. The results of the observation of the worn surfaces are supported by the significantly improved specific wear rate.

### 2.6. Limiting pv Value

**Figure 13:**
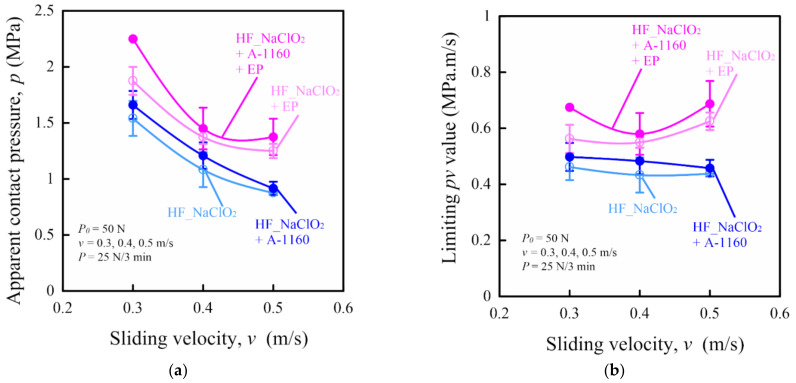
Tribological properties by the step load method of various surface-treated HF/PA1010 biomass composites: (**a**) apparent contact pressure as a function of sliding velocity and (**b**) limiting *pv* value as a function of sliding velocity.

Finally, the limiting *pv* (pressure *p* × velocity *v*) value test results by the step load method using a ring-on-plate-type sliding wear tester of various surface-treated HF/PA1010 biomass composites are discussed. These limiting *pv* values were used as the criteria to quantify the thermal and wear resistance of engineering material for mechanical sliding parts and to evaluate critical operating conditions where the material is unable to bear the load and velocity due to breakage or melting [12,66,67,68]. Figure 13 shows the tribological properties of various surface-treated HF/PA1010 biomass composites investigated by the step load method. The apparent contact pressure *p* as a function of sliding velocity *v* of various surface-treated HF/PA1010 biomass composites is plotted in Figure 13a, which are called *pv* curves, and the limiting *pv* value as a function of sliding velocity *v* of these biomass composites in Figure 13b. The step load (the initial normal load *P_0_* of 50 N and step load *P* of 25 N every 3 min) just before the test piece fractured or melted was defined as the limiting load *P_lim_*. This *P_lim_* was divided by the apparent contact area *A_a_* = 2 cm^2^, and the value obtained was taken as the apparent limiting contact pressure *p*. The limiting *pv* value was calculated by multiplying this *p* and the sliding velocity *v*. In Figure 13a, the apparent contact pressure *p* of various surface-treated HF/PA1010 biomass composites decreased with increasing sliding velocity *v*. The heights of *pv* curves changed with the type of surface treatment and might have increased in some cases in the following order: HF_NaClO_2_ < HF_NaClO_2_ + A-1160 < HF_NaClO_2_ + EP < HF_NaClO_2_ + A-1160 + EP. In Figure 13b, the effects of sliding velocity on the limiting *pv* value of HF_NaClO_2_ and HF_NaClO_2_ + A-1160 can hardly be seen. On the other hand, the limiting *pv* value of HF_NaClO_2_ + EP increased with increasing sliding velocity, and that of HF_NaClO_2_ + A-1160 + EP was minimum at 0.4 m/s. It should especially be noted that HF_NaClO_2_ + A-1160 + EP is the most effective surface-treatment method for improving the limiting *pv* value of HF/PA1010 biomass composites. These tendencies are similar to the bending properties of these biomass composites. These results indicate that these limiting *pv* values are closely related to the load-bearing ability and the resistance to deformation of these biomass composites [12,68]. The limiting *pv* value is considered to be due to the change in the sliding wear mechanisms according to the type of surface treatment of HF, caused by the interfacial interaction between HF and PA1010 and good fiber dispersion in the composites.

## 3. Materials and Methods

### 3.1. Materials

The materials used in this study were epoxy-resin-treated hemp fiber (HF)-reinforced plant-derived polyamide 1010 (PA1010) biomass composites. Plant-derived polyamide 1010 (PA1010, Vestamid, Terra DS16, Daicel Evonic Ltd., Tokyo, Japan) was used as the matrix polymer. PA1010 is a fully bio-based polymer made from sebacic acid and decamethylenediamine obtained from plant-derived castor oil [12]. Hemp fiber (HF, *ϕ*50–100 µm, Hemp Levo Inc., Tokyo, Japan) was used as a reinforcement fiber and was precut into 5 mm pieces. Four types of surface treatments for HF were carried out: (a) alkaline treatment by 5 wt % sodium chlorite solution (NaClO_2_), (b) surface treatment using epoxy resin (bisphenol A-type epoxy resin, jER828, Mitsubishi Chemical Co., Tokyo, Japan) solution after alkaline treatment using NaClO_2_ (NaClO_2_ + EP), (c) surface treatment using a 1 wt % ureidosilane coupling agent (3-ureidopropyltrimethoxy silane, SILQUEST A-1160, Momentive Performance Material Inc., Waterford, NY, USA) solution after alkaline treatment using NaClO_2_ (NaClO_2_ + A-1160), and (d) surface treatment using epoxy resin solution after surface treatment combining NaClO_2_ alkaline treatment and the use of an ureidosilane coupling agent (NaClO_2_ + A-1160 + EP). Epoxy resin (EP) treatment was carried out as follows: 2-butanone solution with a concentration of 1 wt % epoxy resin was placed in a beaker and stirred continuously for 15 min. Then, fibers surface-treated using a combination of NaClO_2_ and A-1160 were immersed in the solution for 60 min. After the treatment, the fibers were removed from the solution and dried in air for 24 h and in a vacuum oven at 80 °C for 5 h. The code, the alkaline treatment, the silane coupling agent, the epoxy resin treatment, and the volume fraction of the fiber *V_f_* of HF/PA1010 biomass composites in this study are listed in Table 2.

### 3.2. Processing

HF/PA1010 biomass composites were extruded using a twin-screw extruder and injection-molded. PA1010 and various surface-treated HFs were dried in a vacuum oven and then dry-blended in a bottle, and subsequently, the melt was mixed at 220 °C and 85 rpm using a twin-screw extruder (TEX30HSS, Japan Steel Works, Ltd., Tokyo, Japan). Test pieces for various experiments were injection-molded by an injection-molding machine (NEX30IV-2EG, Nissei Plastic Industrial, Co. Ltd., Nagano, Japan). The molding conditions were as follows: cylinder temperature of 220 °C, mold (cavity) temperature of 30 °C, and injection rate of 13 cm^3^/s. The test pieces were kept for at least 24 h at 23 °C in desiccators after injection molding to maintain them in a dry condition in all the measurements.

### 3.3. Experimental Method

Fourier-transform infrared spectra of various treated hemp fibers were measured using an FT-IR spectrometer (FT/IR-6100, JASCO Co., Tokyo, Japan) by the attenuated total reflection (ATR) technique using a diamond prism. Spectra were obtained with a total of 64 scans for each sample between 400 cm^−1^ and 4000 cm^−1^, with a resolution of 8 cm^−1^.

Thermogravimetric analysis (TGA) was carried out using TGA equipment (DTG-60, SHIMADZU Co. Ltd., Kyoto, Japan). The samples used for TGA were made by cutting injection-molded coupon specimens into small pieces weighing 10 mg. The temperature of the TGA measurement was set from 50 °C to 400 °C, with a heating rate of 10 °C/min.

Two types of static mechanical property tests were carried out in this study: tensile tests and three-point bending tests. Average values and standard deviations are shown in Figure 5 and Figure 6. Tensile tests (number of samples *n* = 5) were carried out with dog-bone samples (12 mm × 60 mm × 2 mm; length of a parallel part 20 mm) on a universal tester (Strograph V-10, Toyo Seiki Seisaku-Sho, Ltd., Tokyo, Japan). The tests were conducted at a crosshead speed of 50 mm/min and room temperature in accordance with JIS K 7113. The tensile strength *σ_t_*, the tensile modulus *E_t_*, and the elongation at break *ε_t_* were obtained from stress-strain curves.

Three-point bend tests (number of samples *n* = 5) were carried out with coupon samples (12 mm × 62 mm × 3 mm) on the universal tester V-10. The tests were conducted at a crosshead speed of 2 mm/min and room temperature in accordance with JIS K 7171. The bending strength *σ_b_* and the bending modulus *E_b_* were evaluated.

Dynamic mechanical analysis (DMA) was performed using a rheometer (ARES-G2, TA instruments Japan, Inc., Tokyo, Japan) capable of performing linear DMA in the solid state. The samples used for DMA were cut from injection-molded coupon specimens to coupon bar samples (50 mm × 5 mm × 2 mm). The storage modulus *E’* and the loss tangent (tan*δ* = loss modulus *E”*/storage modulus *E’*) of the composites were evaluated as a function of temperature from −100 °C to 220 °C in a nitrogen atmosphere with a constant heating rate of 2 °C/min and tensile fixture at a frequency of 1 Hz. The strain amplitude was set at 0.05%.

Tribological properties were investigated using a ring-on-plate-type sliding wear tester (EFM-III-EN, Orientec, Co. Ltd., Tokyo, Japan) in accordance with JIS 7218a (tested by continuous contact between the test specimens and the metal counterpart (carbon steel S45C)) under room temperature. Two types of tribological tests were carried out in this study: the constant normal load and constant sliding velocity test (number of samples *n* = 3) and the limiting *pv* (pressure *p* × velocity *v*) value test by the step load method (number of samples *n* = 3). Since the experimental methods are the same as the one in our previous articles [12], details are omitted here.

The surfaces of various surface-treated HF/PA1010 biomass composites fractured cryogenically in liquid nitrogen and the polished surfaces of these biomass composites finished with the polishing machine (MI-233-A/J15, Meiwafosis Co. Ltd., Tokyo, Japan) were observed using a scanning electron microscope (SEM; JSM6360LA, JEOL Ltd., Tokyo, Japan) to understand the microstructure in the composites. In addition, the wear debris, the worn surfaces of these biomass polymer composites, and the counterface of the metal counterpart after the sliding wear test were observed using a SEM. The surfaces of all samples were sputter-coated with osmium (Os). In addition, the three-dimensional profiles of the worn surfaces (wear tracks) of these biomass composites were observed using a three-dimensional laser scanning confocal microscope (VK-X200, Keyence Corp., Osaka, Japan).

## 4. Conclusions

We studied the influence of epoxy resin treatment on the mechanical and tribological properties of hemp fiber (HF)-reinforced plant-derived polyamide 1010 (PA1010) biomass composites. The following results were obtained:

1. Mechanical properties such as tensile strength, tensile modulus, bending strength and bending modulus of HF/PA1010 biomass composites improved with surface treatment using epoxy resin.
2. The specific wear rate by the constant normal load and constant sliding velocity test for HF/PA1010 biomass composites significantly improved with surface treatment using epoxy resin, although the frictional coefficient of epoxy-resin-treated HF/PA1010 biomass composites was higher than that of ureidosilane-treated HF/PA1010 biomass composites.
3. The morphologies of the metallic counterface, wear debris, and worn surface differed according to the type of surface treatment carried out. These behaviors may be due to the change in the mode of friction and wear mechanism according to the type of surface treatment, caused by interfacial interaction between HF and PA1010 and fiber dispersion of HF in the composites.
4. The limiting *pv* value by the step load method improved when HFs in HF/PA1010 biomass composites were surface-treated using epoxy resin. These tendencies are similar to the mechanical properties of these biomass composites, such as tensile and bending properties.
5. In particular, pre-treatment combining NaClO_2_ alkaline treatment and the use of an ureidosilane coupling agent (NaClO_2_ + A-1160) is better than NaClO_2_ alkaline treatment alone, before surface treatment using epoxy resin for enhancing the thermal stability, strength, modulus, specific wear rate, and limiting *pv* value of HF/PA1010 biomass composites.
6. In conclusion, surface treatment using epoxy resin after surface treatment with NaClO_2_ alkaline and an ureidosilane coupling agent (NaClO_2_ + A-1160 + EP) is most effective for enhancing the mechanical and tribological properties of HF/PA1010 biomass composites.

## Figures and Tables

**Table 1:** Temperatures at 10 and 15 wt % loss of various surface-treated HF/PA1010 biomass composites.

Temperature (°C)	HF_NaClO_2_	HF_NaClO_2_ + EP	HF_NaClO_2_ + A-1160	HF_NaClO_2_ + A-1160 + EP
10 wt % loss	340.1	342.9	354.1	357.2
15 wt % loss	362.2	362.3	370.4	373.2

**Table 2:** Code, composition, and type of surface treatment of HF/PA1010 biomass composites used in this study.

Code	PA1010 (Vol %)	Hemp Fiber (Vol %)	Alkaline Treatment (NaClO_2_)	Ureidosilane Coupling Agent (A-1160)	Epoxy Resin (EP) Treatment
HF_NaClO_2_	80	20	○	−	−
HF_NaClO_2_ + EP	80	20	○	−	○
HF_NaClO_2_ + A-1160	80	20	○	○	−
HF_NaClO_2_ + A-1160 + EP	80	20	○	○	○

## Data Availability

Not applicable.

## Sample Availability

Samples of the epoxy resin treated HF/PA1010 biomass composites are available from the authors.
